# The Wish to Cure and the Curiosity to Investigate – Or How I Used My Life to Become a Physician-Scientist

**DOI:** 10.3389/fmed.2015.00009

**Published:** 2015-03-06

**Authors:** Jochen Walter Ulrich Fries

**Affiliations:** ^1^Department of Pathology, University Hospital of Köln, Köln, Germany

**Keywords:** physician−scientist, pathology, education, medical, autobiographical, nephrology

## Abstract

The author describes how he became a physician−scientist: difficulties he had to overcome coming from outside of the US (visa, funding, resident training), and his way back to Germany, while experiencing the thrill of actively participating in moving science. Setbacks, scientific success, adaptation to new developments, and the encounter of kindred spirits characterize this lifelong effort.

## The Beginnings

My special interests have always been the heart and kidney – you may say I was put through my paces to become both, a physician (anatomic and surgical pathologist) and a translational researcher. This had three reasons: (i) I always wanted to understand how something evolved rather than just to learn it *by heart*; (ii) I am impressed by tradition, but also eager to learn something new, and (iii) it always appealed to me that my father – as a doctor of internal medicine – could help other people.

The first of these reasons:

(i) Was based on my history class in high school in Wiesbaden, Hessen, Germany. In my mind, I wanted to become an archeologist to explore so many unknown achievements of the antiquities, which have influenced the development of mankind to this day. Fortunately, my parents allowed me to see many ancient places and also modern living conditions in the Eastern Mediterranean and as well as in Middle and South America. This knowledge helped me more than once to bridge cultural and social gaps, and to foster an understanding of different people with whom I later worked.
(ii) My respect for tradition was born while in the boy scouts in a group called “Wilhelmus” and in the Oranienschule (high school – gymnasium) in Wiesbaden, the former capital of old Hessen-Nassau. I admired the fight for freedom by Wilhelm von Nassau – Oranien, the later King of the Netherlands. Nassau-Oranien and its shield with the three golden lilies on a blue ground held a promise to succeed against the odds.
(iii) And medicine? Well, it was my second choice after archeology, and when I finished my “Abitur” (the high school diploma in Germany), things looked rather bleak for classic archeology. Therefore, I did my premedical training and decided medicine was worth a try.

## The Mainz Years

The next event that determined my later fate was the opportunity to visit different preclinical departments at the Johannes-Gutenberg-University of Mainz, where I had started my medical school studies at the age of 18 years. In anatomy, a DNA molecule was made visible by rotary shadowing that was on display in the electron microscope. This was for me the most impressive single event in these 2 years of preclinical medicine. Besides, I liked the insights that I could gain in the biochemistry class, which was taught to us so that we should begin to understand how chemical formulas translated into the meaningful processes regulating life and death.

After passing the so-called “Physikum” the most impressive clinical–theoretical subject was the renal course taught by the new head of the department, Wolfgang Thoenes, M.D., Professor of Pathology. It was all black board and colored chalk, kodachromes were the exception! It was a time with no sonography, CT, or MRI – so autopsy pathology was essential to know from what the patient ultimately was suffering and had died. The daily interactions between the pathologist and the clinical colleagues as they discussed the findings were most enlightening. Over the past 30 years, the average age of the patient has increased by 10 years and now involves treatment of diseases that were unthinkable when I started in 1980. This development increased the complexities of diagnoses and therapies alike, which, in my mind, can hardly anywhere be better evaluated than in a clinical–pathological autopsy conference. The problems of the existing diagnostic tools, the often non-symptomatic and, thus, undiagnosed disease manifestations, and iatrogenic complications have been and are still fascinating to me. Thus, I decided to start with a 1- to 2-year training in Pathology.

I asked for a medical thesis in the Pathology department. I experienced the thrill of being offered to work with a subject, which I had found enticing before: this time it was the electron microscope. 1975 was a time where electron microscopy was the ultimate tool in Pathology. My thesis work took me 4 years to complete up to the end of my medical studies. The microscopic chamber allowed 12 individual pictures to be taken; and I took about 2000 pictures … My mentor was Karl-Heinz Langer, M.D., Professor of Pathology, who was a diligent and skillful experimental pathologist and electron microscopist (1).

## Into Clinical Practice and the Navy

**Figure 1:**
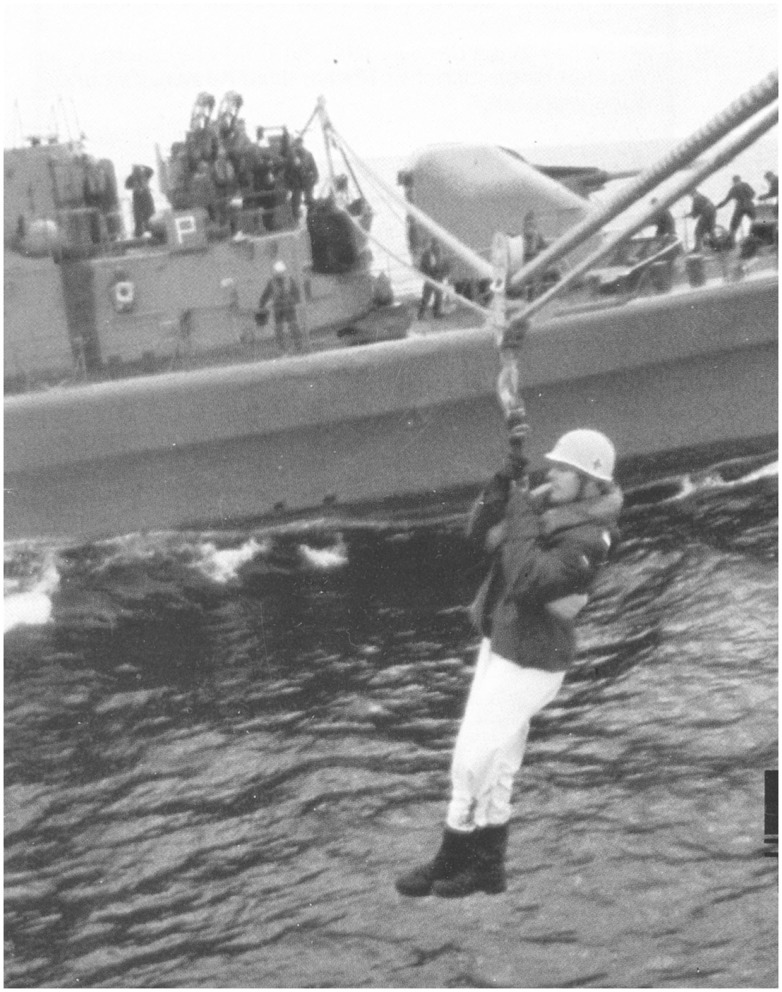
**Sometimes my life depended on a rope …**. Highline maneuver, German Navy, North Sea, 1979.

After an interesting clinical year in the Hospital of Idar-Oberstein, I did my national military service as medical doctor in the rank of a lieutenant commander on board of the German frigate F223, “Karlsruhe” (Figure 1). It was basic medicine at its finest: from pulling teeth, extracting nails, and other kinds of small surgery, including the removal of several small scrotal lipomas with an icing spray as anesthesia (“my wife said, you can do it doctor!”). I learned how to stand up to fight for my patients’ rights when being questioned by a commanding officer. During a three-month trip across the Atlantic, I refused to do a finger amputation (not alone for medical reasons) on a commissioned officer of an English frigate, preserving his gripping function through an operation in the hospital of Halifax in Canada. A diving course in Kiel and a futile search for a rating who went overboard in the wild seas of the Skagerrak in January were further memorable events. After my service, I choose a position as resident in the Pathology department in Mainz, where I had done my doctoral thesis, starting on May 1, 1980.

## As Resident in Mainz, Germany

**Figure 2:**
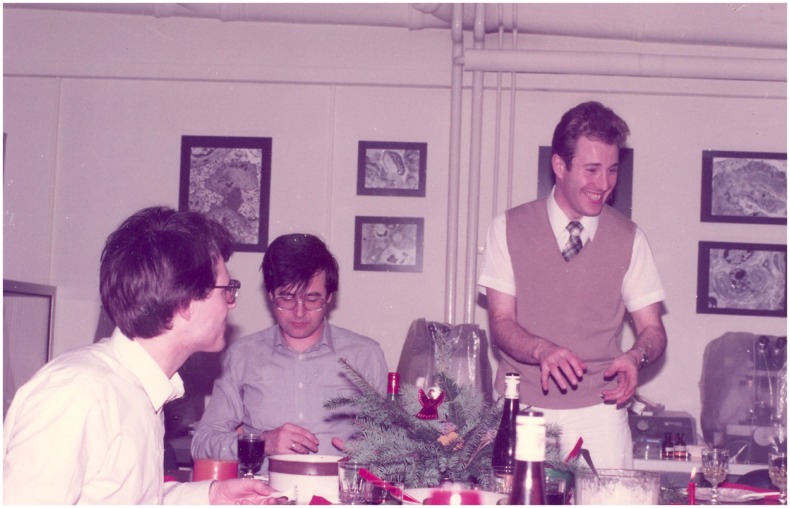
**Advent celebration in good, old tradition in the electron microscopy lab; Department of Pathology, University of Mainz, 1982**.

There was a different understanding at that time of what it meant to strive for a habilitation than today, which Wolfgang Thoenes – a world renowned specialist on glomerulonephritis (GN) – enforced like a code of honor with all his coworkers. The work we did was supposed to help to understand, improve, and set new standards in the chosen field in pathology: such as the development of a renal tumor classification (Stephan Störkel, M.D., Professor of Pathology); the classification of post infectious GN (Karin Sorger, M.D., Professor of Pathology), the classification of membranoproliferative GN (D. Anders, M.D.), micropuncture studies, granuloma formation, and glomerular filtration using Ferritin as tracer (Karl-Heinz Langer, M.D., Professor of Pathology), Alport Syndrome, and renal transplant rejection (Hans-Joachim Rumpelt, M.D., Professor of Pathology). Moreover, to this “code,” I still feel obliged today. Looking back, it was also a time of some nice social interactions (Figure 2).

I stayed in Mainz since I liked the identification of shapes and colors in tissue disease – very similar to the skills that one need for the identification of pottery or pieces of a fries in archeology. My interest in photography came in handy.

Already then, I was intrigued by applying new technology to analyzing “old” problems. The findings in proteinuric disease – particularly the so-called fusion of podocytic processes, being in reality a retraction and flattening of the podocytes – was a pathophysiologically puzzling alteration. Applying a morphometric analysis to a large number of adult and children’s cases in electron microscopic pictures taken by myself (20 capillary loops per glomerulus; 5 glomeruli per case, 5 cases per etiologic group; 5 groups each for adult and children) took me 2 years to finish (mostly in the evenings and at night). I purchased the new hand held calculator by Hewlett Packard; model HP-41C (about 750 DM in 1980) with its newly released statistic module, in order to analyze the enormous amounts of data (and which is still functioning today, 30 years later!). To make matters worse, Dr. Thoenes did not trust the data as much as he trusted his visual evaluation of pictures: how could I propose that only about 10% of all podocytes were flattened and retracted irrespective of the underlying proteinuria and disease entity? – I insisted (“Advent, Advent. Is there a light of hope?” Dr. Thoenes asked me before Christmas of 1986) finally, in 1987 and after much ado, the paper was published in Kidney International (2). Persistence and one’s belief in one’s own data is a lesson one has to learn.

What irritated me a lot then and still does today, however, was and is the persisting belief that experimental research can be done besides medical routine – in the evenings, at weekends, and in your own holidays (as I was told starting as a resident) being not given any protected, extra time, very much in contrast to conditions our American colleagues can enjoy, with fixed times for clinical but also research activities if desired. The major difference, however, is the fact that the NIH pays for the research time, provided one has his or her own (!) grant, while the clinic pays only one’s clinical activities – in contrast to how it is in Germany.

My first talk was given at the pathology meeting taking place each year in Mainz. Since it was a chance for the young residents to get first experiences in an oral presentation, it was charmingly also called “debutant ball.” I had only one night to practice it, while real “pros” practiced their talk once a day for 4 weeks to appear most relaxed …. One is not only dreaming about it, but also having nightmares.

Meanwhile, I had heard about doctors to go abroad to do clinically oriented research in the United States. I realized the enormous potential that such a study might offer. Then, there was that International Meeting in Brussels, Belgium in Oct 1982 about the “Pathogenic Role of Cationic Proteins: Interaction with Biological Membranes.” Scientists were reporting about cell surfaces and their newly discovered negatively charged surface coat. Prof. Thoenes wanted me to go, which was an unexpected honor bestowed upon me not without some envy from other department members (meaning I had to do some extra autopsy work of difficult cases). During the ongoing meeting in the lecture hall, he expected me to simultaneously translate for him. My efforts were to my utmost, but hopelessly inadequate. In the evening cocktail meeting, he asked me suddenly with which of the speakers I would choose to do a postdoctoral training with. I was most impressed by the clearly structured lecture given by Dr. Helmut Rennke, Professor of Pathology at the Brigham and Women’s Hospital (BWH), Harvard Medical School in Boston, MA, USA. The reception ended with me having a chance to personally meet the queen, HRH Fabiola of Belgium, under whose patronage the meeting had taken place. In short, it may pay off having a suit and knowing how to tie a tie properly, if you are a male.

At the farewell dinner, I happened to sit beside Frank Carone, M.D., Professor and Head of Pathology at the University of Chicago. However, far from enjoying myself, I was hard pressed by the wife of the Weitzman scientist to explain the situation in Germany before and after the war. Overall, I must have done well enough to earn an offer to join the Chicago department as a postdoctoral fellow. Prof. Carone supposedly has three beautiful daughters; maybe my life would have been different if Dr. Thoenes had not insisted that I apply to Dr. Rennke’s laboratory at the Harvard Medical School out of prestigious reasons.

In 1983, Dr. Thoenes tried to send me first to Yale University to Dr. Marilyn Farquhar, the scientist being married to Dr. George Palade, who was awarded the Nobel Prize for his innovations in electron microscopy and cell fractionation in 1974. It was the first time I experienced the “uplifting” feeling of being weighed and found to be “too light.” So, I got my “wish” writing to Helmut Rennke in Boston, whom I visited in summer of 1983 to talk about a potential project to propose to the DFG (the German funding organization similar but much smaller than the NIH). When I proposed studying the composition of the immune complexes as a possible cause for their deposition in the glomerulus, my department head did not approve at all – he wanted to know, why protein would go through the capillary wall. Dr. Rennke and others coincidentally, had already studied this, about that time (but with no Dr. Google and PubMed at hand, we did not know). Therefore, my former thesis adviser, Dr. Langer had to travel with me to Boston as my counselor. While we understood each other quite well, I felt that this would not necessarily increase my chances of being accepted.

Unknown to me, in Boston Helmut Rennke had submitted a NIH grant, of which the idea of immune complex location as fundamental cause for the clinical symptoms was a key proposal. In my desperation to answer his question what I wanted to do, I repeated the idea from Mainz: he convinced me that “building” our own immune complexes being located subendothelially or subepithelially would be easier than trying to analyze already formed complexes (which has subsequently been done, too). Coming back to Mainz with Helmut’s proposal, I was not greeted with enthusiasm. On the contrary, I was told that neither scientifically nor personality-wise or in the subject of Pathology could I possibly be successful – so, I should rather leave. This was the “birthday gift” from this department on my 30th. Since Helmut supported me, I was given a 2-year DFG fellowship abroad. A new part of my life started at the BWH, a teaching hospital of the Harvard Medical School, in Boston, MA, USA, on July 1, 1984.

## Post-Doctoral Fellow in Boston: Renal Research

Going to the US I fulfilled a dream of my mother, who was offered training as teacher in the US in 1945, but fell ill with rheumatic fever, convinced that she heard the plane leave overhead, without her. My father was a medic in the Second World War and at the age of 18 years was captured in Monte Casino, Italy, in 1944. He worked as a medic in the POW camp of Fort Devens, a few miles north of Boston. Thus, I carried an invisible burden to succeed.

My level of expectations and fears could have been hardly smaller than that of Columbus starting his first voyage in 1492. My advantage was a much faster and certainly safer travel. I was greeted warmly, and was able to stay at Helmut’s house until he and his newly wed wife had helped me to find my own apartment. In addition, I was equipped with many useful things from other people’s moves coming from his parents-in-law. This way I learned the importance of looking for disposed things in the appropriate room located on each floor of the big apartment buildings: plants, radio, a cheese fondue set (made in Japan!), and clothing forgotten in the dryer, among others. However, prices were high: being paid in German Mark, the exchange rate in 1984 was $1 being about 3 DM: so I had roughly a $1000 to live on. I became good friends with the subway system. That I did not have a car in America was probably the most puzzling news for my friends at home. In spite of having my “own” money, I was expected to work hard, as the chairman of this department, Ramzi Cotran, M.D., Professor of Pathology, communicated to me in no uncertain terms.

It took the better of the first half year alone to establish the appropriate method to position immune complexes by physical and chemical modification. The first animal experiments took me about 4 h per animal; later I could do one in about one half hour (with an assisting hand). I perfused the animals with the differentially charged or sized iron core ferritin molecules in one renal artery. Its deposition was thereby directed within the layers of the peripheral capillary loop followed by antibody injection against ferritin to form an immune complex. Classic pathology techniques (immune histology, electron microscopy), antibody analysis by immunodiffusion, leukocyte detection by chloroacetate esterase staining were among the techniques I learned to employ. This paper (3) was the exact experimental proof of the observations that Dr. Karin Sorger had published in Mainz. It never received quite the attention it may have deserved, since it definitely proved that subendothelial deposition lead to an inflammatory response, while subepithelial deposition lead to proteinuria.

At the time, Helmut collaborated with Barry Brenner, M.D., Professor of Nephrology, and head of the Department of Nephrology, who had become known for his work on hypertensive kidney damage leading to focal–segmental sclerosis (FSGS). He and his fellows were convinced that he should get the Nobel Prize for his discoveries: in anticipation, he had all citations of papers and abstracts printed on watermark paper, with gilded edges and leather bound volumes sorted by year. Thus, I subsequently worked on a second paper, demonstrating by animal experimentation the importance of podocyte damage in addition to forced filtration by hypertension following renal ablation (4).

My funniest scientific experience in these years was getting to the International Conference of Pathology in London, England, in the summer of 1986 to present two posters. Besides meeting my parents, whom I had not seen for a while, I succeeded in getting a stipend paying for the conference. When I suggested the idea of applying to Helmut at first he was not overjoyed – but with the help of a snowstorm preventing him leaving the lab I finally managed to convince him. Furthermore, since the responsible chairperson for the travel grant section was a French scientist, I decided to write my application in French, which I had in school as a third language but I had never used since. Probably, he had a good laugh at the mistakes I made, but it worked – sometimes learning vocabulary can be useful after all. In London, I had to visit the US consulate to extend my J-1 Visa as I was granted an extension by the DFG for one more year. From then on, I was greeted at US customs with increased attention: being a German citizen, staying in the US, but having a J-1 visa from the US consulate in England. I had lots of explaining to do every single time.

There were different research fellows working with Helmut and members of his lab, and I marveled at their determination and skills. However, there is nothing without exceptions. One of the more colorful characters was a female Ph.D., called Jane. Besides cross-country skiing in the yard of the medical school with our male technician on less than an 1″ of snow (rare in Australia, where she came from), she was less skilled in doing experimental work. While harvesting spleens for a fusion experiment to produce monoclonal antibodies, she stood suddenly in the frame of the door of our second lab, where I did an experiment with Helmut’s help. “Guess what chaps, my mouse is on fire.” Touching the mouse with a freshly flamed forceps, she set it ablaze and left it burning although a sink was readily available right behind her. Helmut’s lightning quick moves were comparable to that of Speedy Gonzales. To no one’s surprise, the splenic lymphocytes could not be used.

The thrill of working with Helmut was based on what today would be called translational research: a clinical question (Why did hypertension damage the glomerulus?) was taken into the lab and analyzed by an experimental approach (different animal models of hypertension and therapeutical interception of developing damage). The insights from this research were reapplied to the clinical situation (reducing intraglomerular pressure by ACE inhibitors). This was and still is today the basic idea behind being a clinician–scientist: you understand key problems in the disease to be studied, but are also able to devise an experimental strategy to dissect out these problems, find a solution, and try to apply that to the clinical problem, hoping to improve it.

The waste paper baskets can provide unbelievable treasures of information. I found the invitation to an *in situ* hybridization course at the Tufts University Medical School in Boston (1982) presented with participation of Heinz Höfler, M.D., Professor Pathology, on sabbatical from Graz, Austria, becoming later Chief of Pathology at the University Hospital of Munich. In contrast to the members of my lab at that time, I somehow had the strong feeling of a new dimension opening for many research fields. But, how to do this? I learned about the possibility of changing to an H-Visa, allowing me to stay in the US, provided someone would actually pay me and give me a position in his or her lab.

There was a rather staggering amount of hurdles I had to overcome. Talking to Dr. Cotran, he advised me to inquire about a position in the Department of Tropical Public Health, Harvard School of Public Health, just across the street from the BWH, where I had worked so far. The former department head of pathology, Gustave Dammin, M.D., Professor of Pathology, who was as acting chief present at the first kidney transplantation at BWH in 1954 and being interested in infectious disease, had good connections to this department and recommended that I follow Dr. Cotran’s suggestions. This department had become famous since the late Andrew Spielman, Ph.D., Professor of Tropical Public Health, had identified the tics causing Lyme disease first seen at Martha’s Vineyard, an island off the coast of Massachusetts.

## Post-Doctoral Fellow in Boston: Molecular Mycobacteriology

So, 1 day I went to Dyann Wirth, Ph.D. and Willi Piessens, MD, Professors of Public Health, the first being my future direct supervisor, the latter the future head of the lab I was working in. My project, changed at the last minute (while they worked on malaria, *Leishmania*, and *Wucheria bancrofti*), was the development of DNA probes to identify atypical mycobacteria by PCR reaction. The project was based on a WHO grant, because HIV-infected patients were increasingly identified as simultaneously having other infections. Since the classical mycobacteria causing tuberculosis could only be differentiated from atypical mycobacteria (being only infectious to immune compromised patients) when cultured, a new method of identification was needed – that was my role and my funding. In 1987, PCR was done with different water baths and a clock being set for each step in at least 50 cycles.

Having a week off, I combined recreation at the Florida Keys with serious studying of the basics in molecular biology. At the end, I was at least able to differentiate RNA from DNA: the rest followed in Boston.

**Figure 3:**
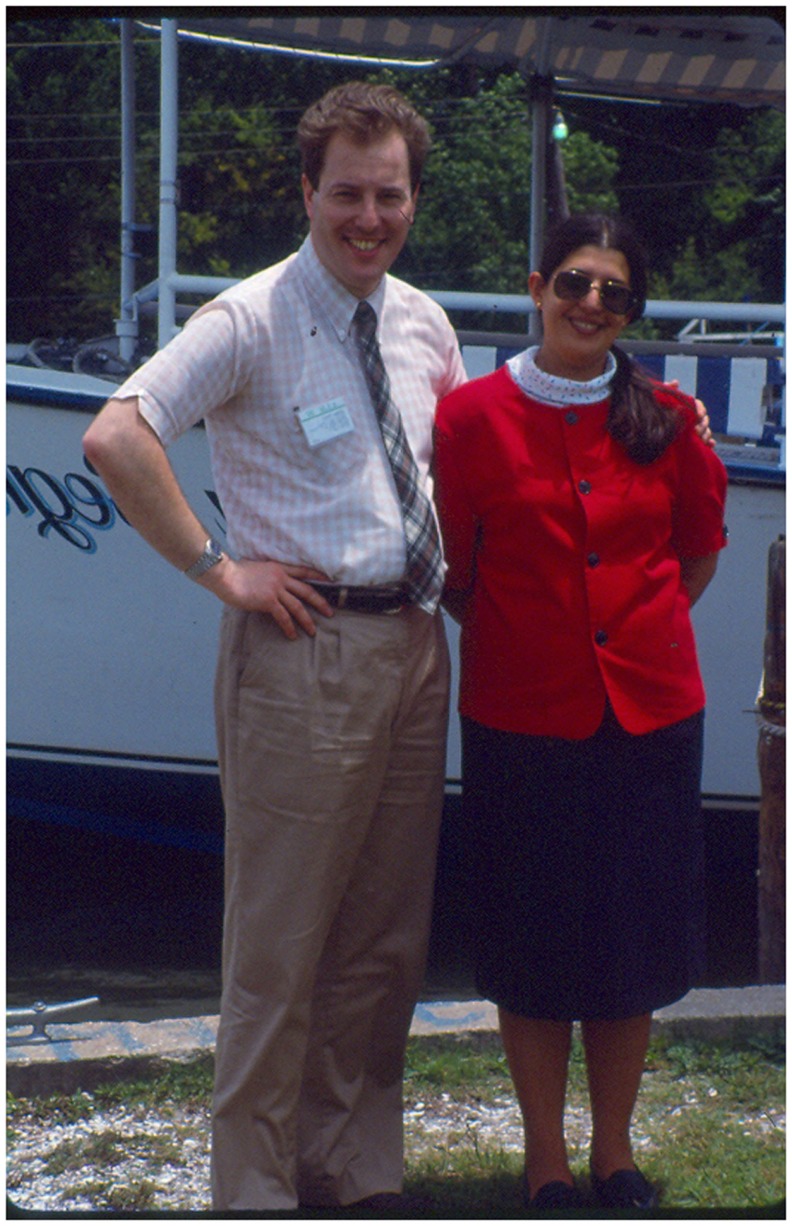
**With Dr. Rubina Patel at the Am Soc of Microbiology Meeting in New Orleans, 1988**.

**Figure 4:**
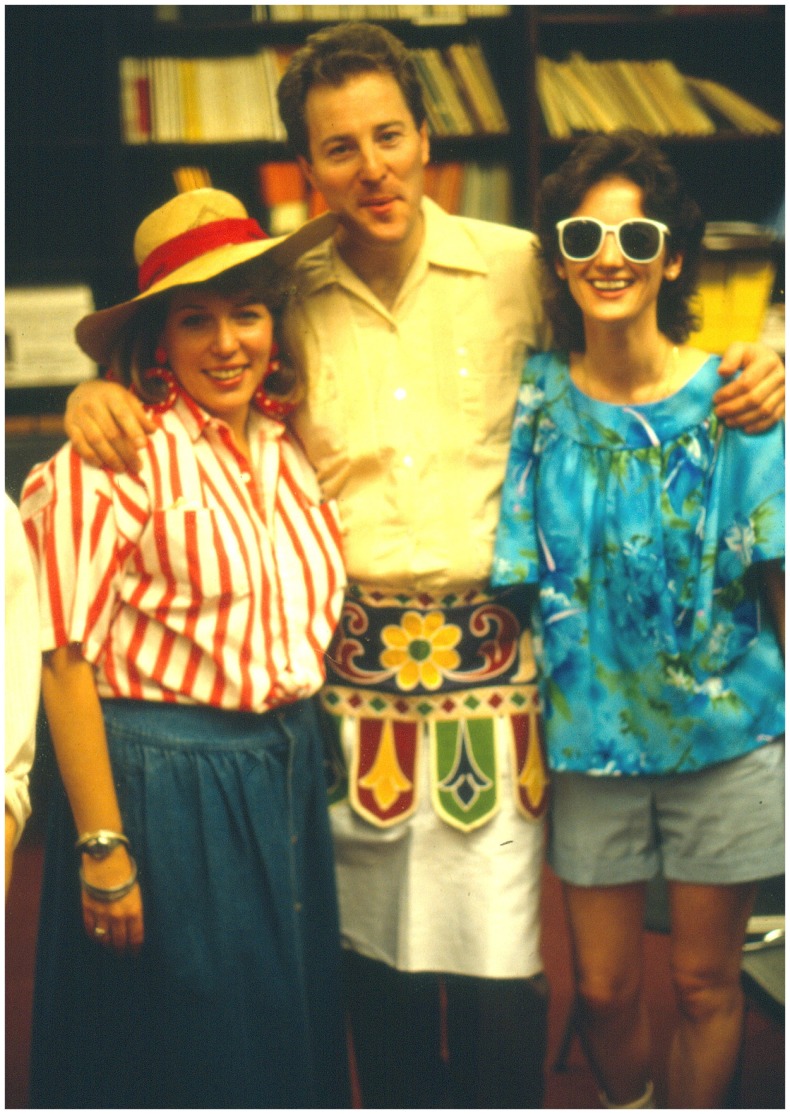
**Celebrating with lab colleagues, Department of Tropical Public Health, Harvard School of Public Health, 1988**.

As fate would have it, I was supposed to take over this project from a female Ph.D., Dr. Rubina Patel, born in Pakistan (Figure 3). Since mycobacterial studies were her scientific life, she was expectedly not pleased by the tactful way this announcement was made, particularly that she had to show me all her techniques and the work in a level 3 laboratory. However, while I was in London, I had the opportunity to visit the British Museum. Here, I had bought a special wedding gift for Rubina. It was just a hunch: I bought a book dealing with ancient places in former Persia, which I had seen on my travels, relating to the Sassanid period and their monotheistic religion founded by Zarathustra. This was indeed her religion, since her ancestors were expelled from Persia to Pakistan long ago. This is how we started our friendship, which was being marveled at by many. Without her help, I would have never ever succeeded in my project. Her meticulous work paired with her always helpful and friendly nature became a lifelong inspiration. When she finally asked me to use one of my buffers, I felt that I had been given a most cherished award. I managed to find specific primers for *M. avium* (5) and developed a PCR protocol to use them on mycobacteria samples from patients (6). These were transported being pre-grown on Lowenstein-Jensen slants in glass (!) tubes from South America to Geneva, where they were picked up by one of our researchers going to a conference, and then flown back to Boston. In case you should wonder about the movies, showing killer bees in the plane, think again: reality can be much more frightening. Overall, it was the most interesting time of my life regarding the different people I met from all over the world (Figure 4). In my final evaluation the negative comment was … . “worries too much about details of experiments”. A comment with whom any scientist can live with.

## On to Instructor in Pathology in Boston: Molecular Cell Adhesion and PDGF

After completing a 2-year and 3 months long, second research fellowship, I wondered what I would be doing in the future. The payment at the School of Public Health was meager. I was not health insured and had no pension payments. My efforts to return to Germany were not successful. Again, I talked to Dr. Cotran. He offered me a position in the lab of Tucker Collins, M.D., Ph.D., Professor of Pathology, at the Vascular Research Division at the BWH, led by Michel Gimbrone, M.D., Professor of Pathology. When I talked to Tucker, as everyone called him, he was impressed by my knowledge of animal experimentation but also by the quality of my sequencing gels. In the fall of 1987, I became part of his lab. He had spearheaded the generation of a transgenic mouse with the PDGF – B-chain promoter – β-galactosidase as transgene to study endothelial cell proliferation. Therefore, he entrusted me with the first transgenic mouse at the BWH to be analyzed. It took me the better part of 2 years to characterize and breed three transgenic mouse lines with different number of transgene copies in their genome. Luckily, I had seen something of Boston and the vicinity before this fellowship started, because afterwards free time, even on the weekends, was very scarce. To my disadvantage, the transgene was not expressed in the endothelium but in the neurons of brain and spinal cord. At the FASEB meeting in 1990, I presented my data (so far unpublished), which consisted of many CAT assays and immune histology showing the expression of the transgene in different brain regions, and in development. When we wanted to publish our paper back to back with that of Elaine Raines (PDGF in the brain of Macaque monkeys) in Cell, I was promised a first authorship. The disappointment was considerable, when we learned that a competing group doing *in situ* hybridization had used the knowledge I presented to push through a paper within 3 months – and got it accepted in Cell. Our paper was accepted as a joint effort with Drs. Raines and Ross (7). The lesson learned from this for me was never to talk about “hot” data at a meeting, which was not accepted for publication before. The even bigger disappointment was the rejection of this animal model for a patent by the BWH, while a couple years later it became one of the favored models for Alzheimer disease by using the PDGF promoter to target neurons. Further experiments using this model and others to study epilepsy took up another year of my time, but never saw the light of day, since PDGF expression in neuron outgrowth following seizures were falsely thought to be neglectable by the advisory neuropathologist at the time. I still see myself sitting in the Boston subway car in summer at almost 100°F with the mouse hippocampi on dry ice, which I had dissected at a collaborating Boston college lab to bring back to my lab to extract RNA for a Northern blot! At least, our promoter study, where I characterized the importance of the ETS-class of transcription factors for the PDGF – B chain expression, was published in JBC in 1994 (8). Meanwhile, I had turned to cloning and characterization of cell adhesion molecules, like the mouse E-selectin and the rat VCAM-1 (vascular cell adhesion molecule-1) (9). Working together with Myron Cybulsky, M.D., a *post doc* at Dr. Gimbrone’s lab, and today Professor of Laboratory Medicine and Pathobiology at the Toronto General Hospital/Research Institute (UHN), we analyzed the functional importance of an alternatively spliced VCAM – molecule present in endothelial cells (10, 11). I was granted a Green Card by BWH and an Instructor of Pathology (the lowest faculty position) in 1992. I was also made a co-author of an NIH grant by Tucker. In my new position as instructor, I did a project with Mary Lipscomb, M.D., the head-to-be of Pathology from the University of Texas Southwestern Medical Center, Dallas, TX, USA. Mary and I understood each other quite well, while we had to adjust to each other’s version of spoken English (hers being with a Southern accent, mine being more German-British). My role was to introduce her to techniques like RNA extraction, frozen section immune histology, and others. Together, we characterized the expression of the rat VCAM-1 and E-selectin (12). How fruitful collaboration can be, was also shown by our work with the immunology lab of Laurie Glimcher, Ph.D., at the Harvard School of Public Health. With my work of E-selectin, I could help to demonstrate that transcription factors may act together in achieving transcriptional initiation (13).

One single event stands out during my stay in Boston: in 1990, I was part of the long line of people being able to congratulate Joseph Murray, M.D., Emeritus Professor of Surgery, on his Nobel Prize in medicine for the first successful kidney transplantation in 1954 at the BWH. He had to fight for many years before being accepted, as he said in an interview in 2004, being harassed as “playing God, disturbing the course of nature or experimenting on human beings.” In 2014, he died at age 93 years, being an advocate of stem cell research. It is consoling to know that it may pay off never to give up on something one believes in.

## Residency at the Brigham and Women’s and at Children’s Hospital, Boston

Eventually, I was asked by Dr. Cotran, whether I wanted to do a residency at the Brigham in Pathology. Just as it is in Germany, the US authorities did not accept my medical training and I had to repeat (and pass) the medical examines (USMLE) and pass a language test before being accepted. It was consoling to learn that the Krebs cycle was still in existence, and the anatomy of the heart had not changed in the15 years since I passed my German boards.

**Figure 5:**
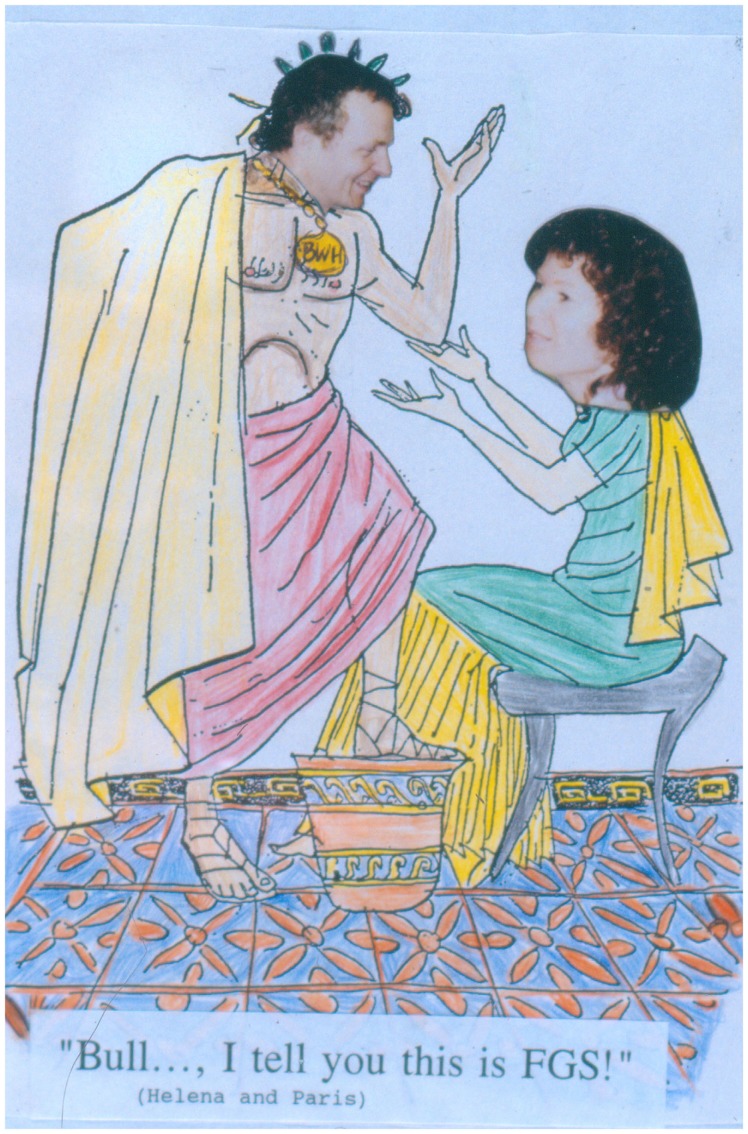
**Making fun of our seniors in pathology at the end-of-the-year party: “Persuasion is a virtue of gods.”** Dr. Helmut Rennke and a resident discussing renal pathology, 1986.

On January 2, 1993, my first day as resident, I started with the disheartening news that my wife, Anne, had lost our first child, was another day never to forget. Luckily, we still managed to have two wonderful children thereafter. Otherwise, residency was a strenuous but interesting time. I liked the structured way it was organized, starting with the beginning of the academic year on July 1, ending on June 30 the following year. We were 20 residents and the schedule was made ahead for the entire year and working like a clock (but only with 3 weeks of vacation!). The institution of a chief resident helping the first year residents to get started successfully was excellent. Additionally, specialties of cardiac, renal, lung, and later sarcoma pathology could be chosen in the second and third year of a 4-year training. Thus, it was not “learning by doing” only, as I experienced later in Germany, but a real one-on-one training with the respective senior pathologist, of which we had about 40. Signing out cases on a Saturday morning from 08:00 to 14:00o’clock was a regular event (being hit by about 200+ glass slides on Friday afternoon to prepare), as were weekend duties for residents. We did our frozen sections by ourselves (not the technicians!): nothing is more exhilarating than a surgeon breathing down your neck, while you try to cut this miserable piece of tissue. However, if you are successful, this will bring a very different and more constructive atmosphere of working together and respecting each other than if the interaction is only done by phone. Single rooms were given only to those board certified pathologists – in other words, training was much harder than in Germany but also far more efficient, team oriented, and much more fun! In addition, our end-of-the-year party was always original and funny (Figure 5).

Ramzi Cotran made every effort that residents were exposed to different research alternatives since he expected them to become clinician–scientists and later heads of departments, training other people in succession, being particularly able to raise interest in research coming from clinical questions (translational research) combined with teaching. Already in our first year, we were to present a case at the so-called Gross-Micro Conference every 2 months: an autopsy case presented from which a scientific problem was deduced, and presented using your own or data from the literature to the pathology audience. Many residents did subsequent postdoctoral work in one of the many experimental labs. The department was well funded; figures reached about eight Million US$ per year.

However, we had also fun in our department and we could even make fun of Ramzi Cotran. Though it may seem that funding was ample, fighting to get a NIH grant through was a very difficult task with temporarily only <10% of all applied grants being funded. For example, at a Christmas party, one of the junior staff members acting as Santa Claus was wondering aloud, why Ramzi Cotran – being otherwise a very diplomatic person – should have advised his senior researchers to behave rather aggressively toward the NIH. Our department head had finished a sabbatical in Leiden, at that time the center of European kidney transplants. Santa Claus presented pictures of him, however, in the pose of Rambo (putting his head on that of Sylvester Stallone) and suggesting that the unexplainable change of attitude came from him being trained as special force soldier (however, not in Leiden!). Dr. Cotran would sign several copies of this picture, of which some ended up on mantelpieces in the respective private homes among traditional pictures of family members.

I developed a research project with Tucker’s permission (meaning he diverted money so that I could finance my own project). I published two papers on the *in vitro* and (for the first time) *in vivo* regulation of the NF-κB signaling pathway in the kidneys of mice. In the attempt to be acceptable for a so-called first award (a first NIH grant for junior research members) I developed and worked on these projects together with another postdoc, Levon Kashigian, Ph.D., today a good friend and currently Professor and Director of the Centre for Vascular Research at the University of South Wales, Sydney, NSW, Australia, and Head of the Transcription and Gene Targeting Group. His encouragement was quite important and carried me through some tough times. On these two papers, I was last author – a prerequisite for the grant, and a great gesture from Tucker (14, 15). However, in Germany, these two papers did not count toward my habilitation (only first authorship) and cost me 2 more years toward finally fulfilling the required 10 first author publications in Köln (nowadays only 5 total!), preventing me from applying for a professorship at a different university successfully.

**Figure 6:**
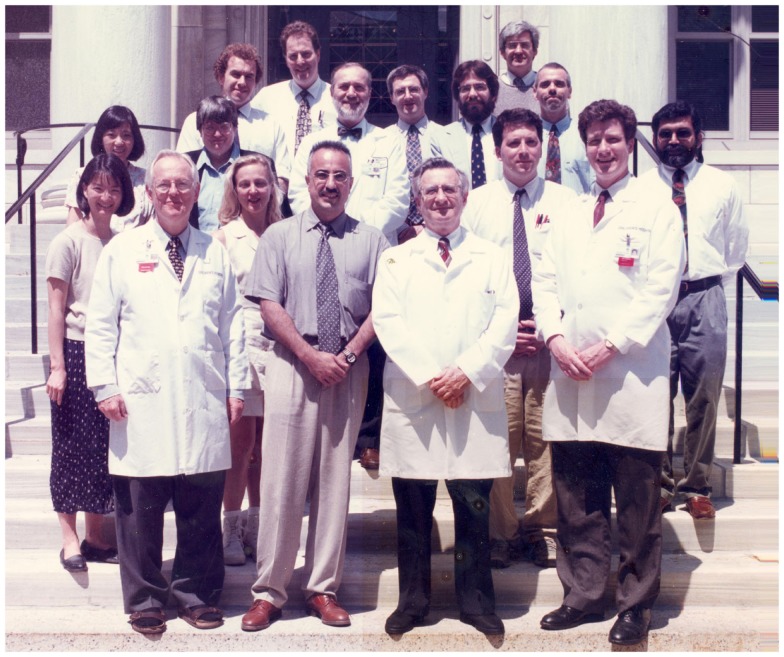
**Staff and residents at the Pathology Department, Children’s Hospital, Boston, 1994: in front (left to right) Dr. van Praagh, Dr. Perez, Dr. Cotran**. I am standing in the last row to the very left.

I decided to do Pediatric Pathology as a specialty. Therefore, I worked a total of 3 years at Boston Children’s Hospital, where Dr. Cotran was also the head of this pathology department (Figure 6). In my third and last year, I worked with Richard van Praagh, M.D., and his wife Stella, M.D., both Professors of Pediatric Cardiology and international experts on congenital heart disease. During this time, I also worked part time as a paid adviser for experimental research in the laboratory of José Halperin, M.D., Professor of Hematology, Laboratory of Translational Research at the Harvard Medical School. We set up the model of Lewis Lung Carcinoma, worked on evaluating arteriosclerosis by morphometry, and tried to intercept scar tissue formation and inflammation, where I used my expertise with the vascular cell adhesion molecule 1.

## The Way Back to Germany: Köln

With my training as a resident ending, I had to decide whether I would consider coming back to Germany. With BWH and the Massachusetts General Hospital expected to merge (“Partners”) – which from today’s standpoint (2014) never happened to the degree originally planned – the options were limited, and my first award (NIH grant for young researchers) needed at least another round of evaluation, thus another year even if it was eventually funded. Meanwhile, I had an offer from the new department in Köln, Hans-Peter Dienes, M.D. This was my last chance for a ticket home, being 44 years of age in 1997. The 13 years in Boston were very fruitful but were not appreciated in Germany (quote: “Without that time in the US, you would have reached your habilitation reasonably early”), and again, they did not count as experience for my pathology position nor for my pension funds! In addition, I had to repeat pathology training, pass my German pathology boards in 2000, my specialty boards in molecular pathology in 2001, and finally my habilitation in 2003. As it was custom in 2002 for the habilitation process, I had to prepare 23 Leitz folders containing copies of all papers and lists of publications and talks, neatly arranged in easily identifiable sections. The copying alone cost more than a 1000 DM. Then, I packed all the folders in a big shopping cart and moved through the entire university hospital to distribute one each per department (in the subsequent year, CDs were introduced as means of presenting data). The respective secretaries greeted me with different degrees of enthusiasm. The interest was limited regarding the subject of my habilitation thesis (“Vascular cell adhesion molecule-1: molecular biologic and molecular pathologic studies regarding its role as vascular and mesangial marker of inflammation”). The review process in my case was reasonably speedy (5 months) except for the neurosurgery department, where – maybe due to a secret admirer – the folder got lost.

**Figure 7:**
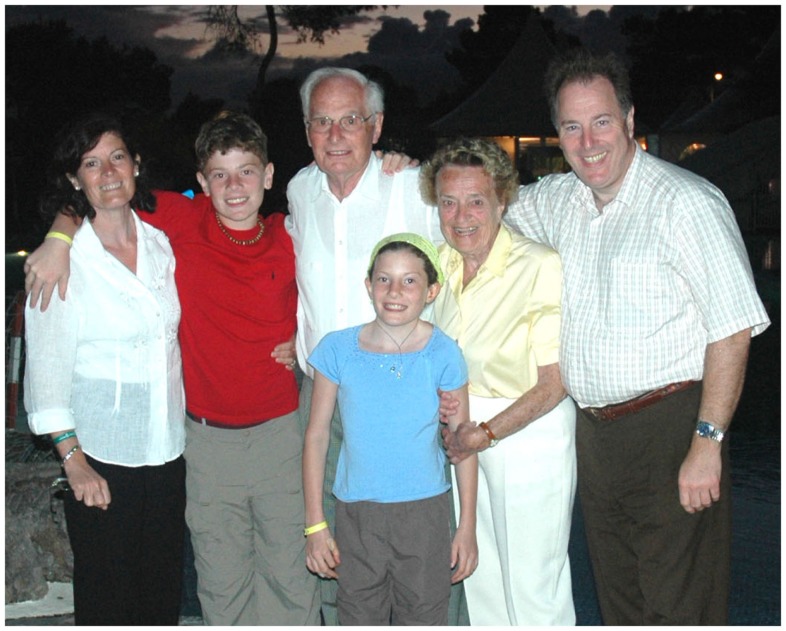
**Family and parents (2006) – the only constant support**.

Was it all worth it? The question to ask yourself, in my mind, however, is why do you want to do basic/translational research as a clinician in the first place? To call yourself a professor: if you desire the title only for your letter head, then this ordeal – even if it is shorter than mine – is not for you, and you may be better served doing clinical studies. Yet, I believe one should try to make the best out of one’s God given gifts. Moreover, if you should have the desire to contribute another piece to the eternal puzzle of mankind, possibly even of some persistent importance, it is definitely worth a try. However, it helps, when your family is on your side …. (Figure 7).

## The Era of Molecular Technology

Thus, being back in Germany – besides trying to overcome all hurdles including a new beginning for my family – there were again several new techniques in the last 10 years, which changed the way, we do research today: laser-microdissection, RNA isolation from fresh and from formalin-fixed, paraffinized tissue, siRNA, microarray technology, and miRNA. In Harvard, I had learned that as soon as a new technology appeared on the horizon, one has to think about how it could be useful for one’s research. As Tucker told me, one has options: you lead, you follow, or you get out of the way. Whether you actually have this choice, and which one you will take in each separate case, may well depend on a variety of factors, unfortunately, not always under your own control. However, do not use it just as an excuse – ask yourself what will become of your plans in the long run.

With my past experience, I was able to get a DFG grant and established laser-microdissection combined with mRNA analysis using femtograms of RNA from paraffinized renal biopsies out of glomerulus-slices (16). All I had was an eager technician fresh from school, Ms. Tanja Roth, whom I taught in my vacation time to do ELISA analysis and RT-PCR. In collaboration with the center of nephropathology at Ljubljana, Slovenia, we collaborated and developed a technique to transfer frozen sections at mid-summer temperatures to Köln in order to extract RNA from glomerular slices for RT-PCR. The yield of RNA from frozen section versus paraffinized tissue was almost a thousand-fold higher. All that, however, was not good enough for the DFG reviewer, a tumor researcher who worked with milligrams of RNA, having no clue about the problems I had to face. I was not happy …. In addition, I tried to publish a paper showing laser-microdissected glomeruli from a lupus patient, showing that in remission previously upregulated genes were no longer detectable. It was not appealing to the renal pathology community. A “real” pathologist would be able to see changes in the microscope. However, changes on gene levels one cannot appreciate by microscopy until they are developing into visible damage. It was this pre-damage analysis I was interested in because it may potentially guide preventive therapy. I felt I was doing the right thing at too early a time point.

In order not to forget my endothelial roots I managed to collaborate with the lab of Claudia Gottstein, Ph.D., Clinic I, Internal Medicine at the University of Köln working on tumor endothelium to induce thrombosis, ultimately using the vascular cell adhesion molecule-1 as target molecule (17, 18).

## The Stem Cell Euphoria

In the first years of the new millenium, the excitement about stem cell research healing disease was very high, spearheaded by the attempt to heal myocardial infarction. My efforts to participate in this development were successful through collaborations with several clinician–scientists from other departments. By Bernd K. Fleischmann, M.D., Professor of Physiology, I was engaged to look at the effects of stem cell infusion in infarcted areas of the mouse heart (19). The desired healing through rebuilding or mitoses of the cardiac myocytes was not observed. When omnipotent stem cells were used, teratomas ensued typically showing differentiation from all germ layers, while pluripotent stem cells predetermined by an expression vector for smooth muscle actin did not (20). Similar results were observed when I collaborated with Jochen Müller-Ehmsen, M.D., in rat hearts with myocardial infarction tracing the injected stem cells by RT-PCR for the Y-chromosome (21) or looking at bone marrow cell deposition in a pig model after balloon occlusion of its coronary arteries (22). Another study with Manfred Gessler, M.D., Professor of Cardiac Surgery, looking at lymphangiogenesis in failing hearts suggested that appositional growth of initial lymphatics, rather than “*de novo*” genesis from pluripotent stem cells or sprouting from preexisting venous vessels, may be the predominant mechanism (23, 24). With all the excitement about the heart, I tried to apply this idea to the kidney, too. Here, I participated in a project led by Volker Burst, M.D., a clinician-scientist from our Department of Nephrology (25). We were looking at the survival and the effect of mesenchymal stem cells repopulating necrotic tubules following acute renal failure in the rat, my model from my doctoral thesis. In agreement with the findings in the heart, the mesenchymal cells were not present long enough and not in sufficient numbers to be able to play a decisive role in the repair of tubular damage.

Another important collaboration showed the importance of mechanical stretch for activation of human mesangial cells from the glomerulus as it happens in hypertension. With Margarete Odenthal, Ph.D., and her group from our department, I performed experiments showing the importance of individual transcription factors for the promoter activation of smooth muscle actin, a marker gene of mesenchymal transition (26).

## A New Focus: Endothelin-1

About 2003, just being finished with my habilitation I was lucky to meet a clinician (pediatric nephrologist) – researcher from our Pediatric department, Christoph Licht, M.D. (today Associate Professor of Pediatrics, Hospital of Sick Children, Toronto, ON, Canada), working on renal damage in children. He had done a postdoc working with Masashi Yanigasawa, M.D., Ph.D., a former investigator at the Howard Hughes Medical Institute and a Professor at the University of Texas Medical Center, on the autocrine role for endothelin-1 in the regulation of proximal tubule NHE3. I assisted him in evaluating a proteinuric model of renal disease being treated with endothelin-receptor blockers versus the classical treatment of ACE inhibitors. Doing this, I remembered that Tucker had once helped a fellow of Dr. Brenner to clone rabbit endothelin-1 in 1992. Therefore – with the delay of almost 14 years – I started a success story investigating endothelin-1with respect to renal tubular disease.

Looking for a Ph.D. candidate to work with me on this project, a colleague referred me to Melanie von Brandenstein, in 2006 a young Master graduate from the Rhein-Bonn Sieg University of Applied Sciences in St. Augustin, German. First, we demonstrated the yet unknown existence of the endothelin A-receptor on human proximal renal tubule cells (27), leading to the activation of the NF-κB inflammatory signal cascade (28). Then, we established a novel signaling pathway, showing that in renal tubule cells as well as in various tumor cell lines, the p65 component of NF-κB, and the p38α component of the mitogen-activated kinase p38 together (29) are joined by the α isoform of protein kinase C to establish a cytoplasmic complex. This complex can transmigrate into the nucleus, where PKCα interacts with the pri-miRNA15a to prevent miRNA maturation. After stimulation, the complex is reduced, less PKCα present in the nucleus, and mature miRNA5a is made (30). Presenting that research at the International Conference of Endothelin in Cambridge in 2011, with my support Melanie was awarded the best presentation and the best poster award. Showing that miRNA15a was excreted in the urine, we demonstrated that miRNA15a could be a urinary biomarker for malignant renal cell carcinoma [von Brandenstein et al. (30)]. This research received the annual award of the Walter Schultz Stiftung, München, in 2012.

**Figure 8:**
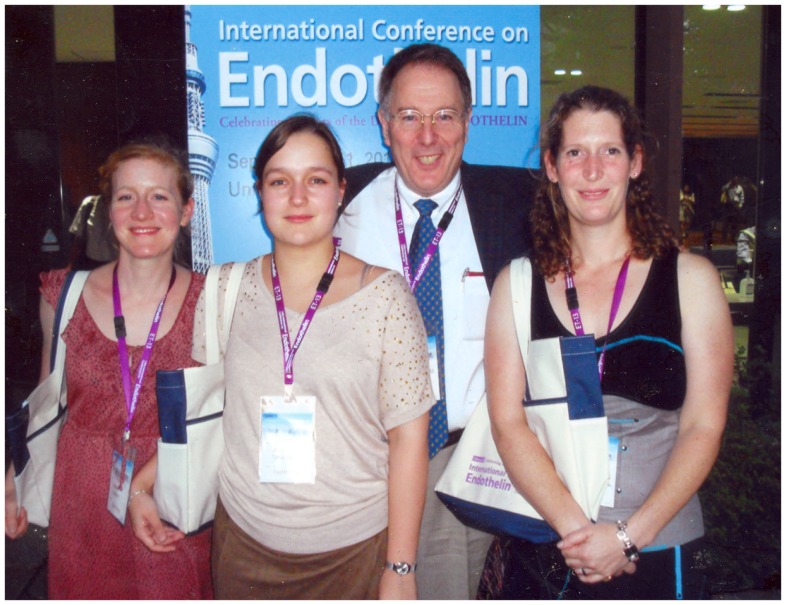
**With Heike Löser, M.D., Julia Straube, BA(!) and Melanie von Brandenstein, Ph.D. (left to right) at the Thirteenth International Conference on Endothelin (ET-13) in Tokyo, 2013, celebrating 25 years of the discovery of endothelin-1**.

To build an international reputation by trying to answer clinically related questions with basic science methods, which was my understanding of doing translational research, is difficult when less and less time can be allotted to do research activities. Hereby, the responsibility for the lab members is a burden not to be underestimated. In 2013, I was finally granted my professorship after all, which opened new avenues for independent research. At the same year, we celebrated the 25th birthday of endothelin in Tokyo (Figure 8).

## With 60 – Ahead into the Future

In January 2014, turning 60, I received the remark that I will probably be looking forward to my retirement now. I also experienced a 30-year-old resident telling me sadly that she still was looking at 35 years of work ahead – what do you think my answer was? Give me your 35 years!! And why should I stop? On the contrary, there are so many exciting projects! Recently, we managed to get a patent on an antibody recognizing a spliced variant of vimentin. Furthermore, I think that we have solved the puzzle of unexplainable vimentin positivity in benign tumors due to the undiscriminating use of commercially available antibodies recognizing only the full length versus the sliced variant, in addition. This insight is very important in the diagnostic field of uropathology, where misdiagnosis of malignant tumors as benign carries potentially grave consequences for the patient. Thus, with Dr. Stefan Störkel, who developed the classification of renal tumors, we are looking currently to enlarge our diagnostic, preoperative potential by finding new urinary biomarkers for other renal tumors. With Heike Löser, M.D., a pathology resident having four (!) children, I submitted a paper describing endothelin-mediated downregulation of a multiple-drug resistant protein due to a miRNA overexpression, leading to tubular damage (31). Besides all of this, who would marvel at the daily gall bladders and appendices more than I?

So, if you consider becoming a clinician–scientist and have still some questions, you may want to talk to me. I would be proud to be one of your mentors helping you discover the thrill of being a physician–scientist, and to improve our scientific understanding of clinical problems. You need to bring endurance, an inquisitive mind, and a good portion of imagination. And most important of all you have to be willing to think outside the box. There may be hardly a project in which you will not be willing to give up in between. If you persist you will eventually succeed, be strengthened, and invigorated for your next project to come.

## Conflict of Interest Statement

The author declares that the research was conducted in the absence of any commercial or financial relationships that could be construed as a potential conflict of interest.
